# Acceptability of at-home urine self-testing for bladder health problems: a qualitative study

**DOI:** 10.1186/s12889-026-28938-1

**Published:** 2026-08-12

**Authors:** Emma Lidington, Ninian Schmeising-Barnes, Megan Goff, Abigail Carter, Phil Kelly, Allen Knight, Eleni Josephides, Marcus Cumberbatch, Peter Sasieni, James WF Catto, Jo Waller

**Affiliations:** 1 Centre for Cancer Screening, Prevention and Early Diagnosis, Queen Mary University of London, London, UK; 2 School of Cancer and Pharmaceutical Sciences, Faculty of Life Sciences and Medicine, King’s College London, London, UK; 3 Patient and Trustee, Action Bladder Cancer, Tetbury, UK; 4 Guy’s and St Thomas’ NHS Foundation Trust, London, UK; 5 Comprehensive Cancer Centre, King’s College London, London, UK; 6 Division of Clinical Medicine, School of Medicine & Population Health, University of Sheffield, Sheffield, UK; 7 Department of Urology, Sheffield Teaching Hospitals NHS Trust, Sheffield, UK

**Keywords:** Acceptability, Cancer screening, Bladder cancer, Haematuria, At-home testing

## Abstract

**Background:**

Haematuria can be a sign of bladder cancer. The YORKSURe feasibility study assessed at-home haematuria dipstick self-testing to inform future effectiveness trials of screening for bladder cancer. Potential participants were mailed self-testing kits and asked to undertake 2–6 dipstick tests. Participants reported results using an app to send the image to the trial team or by phone (matching their test strip colour to a chart and entering the corresponding number). The dipstick tested five parameters and participants were randomised to reveal different panel outcomes. Those with predefined abnormal results were offered further investigation in community clinics. We qualitatively explored the acceptability of urine self-testing, self-reporting results, follow-up tests and the trial design (including revealing/concealing results) in addition to participants’ experience receiving the results and barriers and facilitators to the test.

**Methods:**

We carried out semi-structured interviews, informed by the Theoretical Framework of Acceptability. We purposively sampled responders and non-responders by participation, test result and socioeconomic deprivation. We analysed verbatim transcripts thematically using the Framework method.

**Results:**

We interviewed 29 people: 21 responders, 6 non-responders, and 2 people who attended an early detection clinic following abnormal dipstick findings. The idea of urine self-testing was acceptable. Many people felt capable of collecting and testing their urine and reporting the results. For others, the stipulations required to collect the urine increased the perceived complexity and the need to self-report the results made the test feel burdensome. Results were understood though some people expected more than one result. Follow-up tests for abnormal results were expected. The most common barrier to testing was timing of the invitation. The most common facilitators were interest in health and past health experiences. There were mixed views on the acceptability of concealing results and concerns around concealing abnormal results.

**Conclusions:**

On balance, most people found the kit and self-reporting acceptable. Simplifying instructions could improve acceptability. Concealment could be further explained in any future trial.

**Clinical trial registration:**

YORKSURe was registered with the ISRCTN on 09 June 2022 under Registration Number 34273159.

**Supplementary Information:**

The online version contains supplementary material available at https://doi.org/10.1186/s12889-026-28938-1.

## Background

Bladder cancer (BC) is a common malignancy with regional patterns of incidence and mortality [1, 2], reflecting risk factor exposures [3–5]. Although earlier diagnosis can be associated with improved survival, no screening programmes currently exist for BC [6]. Most BCs are diagnosed in people with visible and non-visible haematuria [7, 8]. However, haematuria is often invisible or only present in the urine intermittently, making screening using single tests unreliable.^9^ Home-testing for haematuria over multiple days offers a potential solution and might help detect BC earlier than otherwise possible [10, 11]. Due to the high prevalence of haematuria and low incidence of BC, population-wide screening could lead to large numbers of people requiring investigation for haematuria (needing cystoscopy and upper urinary tract imaging) without impacting BC survival [12]. A targeted approach to enrich the screened population, such as smoking status and environmental exposures, could reduce the extent of over screening [13].

The YORKSURe study is examining the feasibility of at-home urine testing for BC in Yorkshire (in the North of England), a region with higher than national average BC mortality rates [14]. The study posted urine self-testing kits to two asymptomatic cohorts of individuals with potential risk factors for BC. Participants were asked to complete the test daily for six days and report their results by phone or mobile app. The test strip included five parameters, but only one parameter was reviewed by a nurse and revealed to the participant. This reveal/conceal design has statistical benefits and ensures all participants receive potentially beneficial information, avoiding the need for a placebo control group. In Cohort 1, all participants received their haematuria result. In Cohort 2, participants were randomly allocated to either receive their haematuria or glycosuria result. Participants with haematuria were further triaged using urine cytology and urinary tract ultrasound. Those with glycosuria were invited for a blood test to investigate for diabetes.

To evaluate the feasibility of this self-testing and self-reporting approach, the study team assessed perceived acceptability alongside process measures, test performance and clinical outcomes. Acceptability has been set out as a key criterion for new screening programmes by the UK National Screening Committee [15]. This includes evidence of the acceptability of the test, result delivery, diagnostic procedures and treatments to the public and healthcare professionals.

A previous study among paediatric and young adult nephrology patients found that at-home urinalysis with a mobile app to scan results was significantly preferred over bringing a urine sample to clinic [16]. Participants appreciated the ease of use, convenience and immediacy of results, although some experienced technical issues. Another study looking at home urine testing for proteinuria among pregnant women with manual colour matching found 96% of them would be willing to check their urine at home. However, qualitative results suggested that colour changes on the strip were sometimes difficult to interpret, some people may not feel capable of interpreting the colour changes and others had concerns about forgetting to do the test or mis-reporting results [17].

This paper describes the qualitative work undertaken to explore the range of views related to the acceptability of urine self-testing, self-reporting results, follow-up investigations in local clinics and the trial design. In addition, we looked at participants’ experience of receiving the self-testing results and barriers and facilitators to doing the test.

## Methods

This was a qualitative sub-study using in-depth, semi-structured interviews.

### Participants

Potential participants included anyone invited to Cohorts 1 or 2 of the YORKSURe study. Full eligibility is described in the YORKSURe protocol [14]. In brief, Cohort 1 included men and women aged 55–80 from the Yorkshire Lung Screening Trial who consented to future contact [18]. Cohort 2 included men only, aged 65–79 in high deprivation areas in South Yorkshire. For this qualitative study, we purposively sampled from these two cohorts by study participation (i.e. whether they chose to participate in YORKSURe or not), clinical outcome and index of multiple deprivation decile (Additional file 1). We planned to conduct at least 20 interviews, continuing recruitment until information saturation was reached (adequate coverage of the charted data in each section of the analytical framework) or resources ran out [19].

**Fig. 1:**
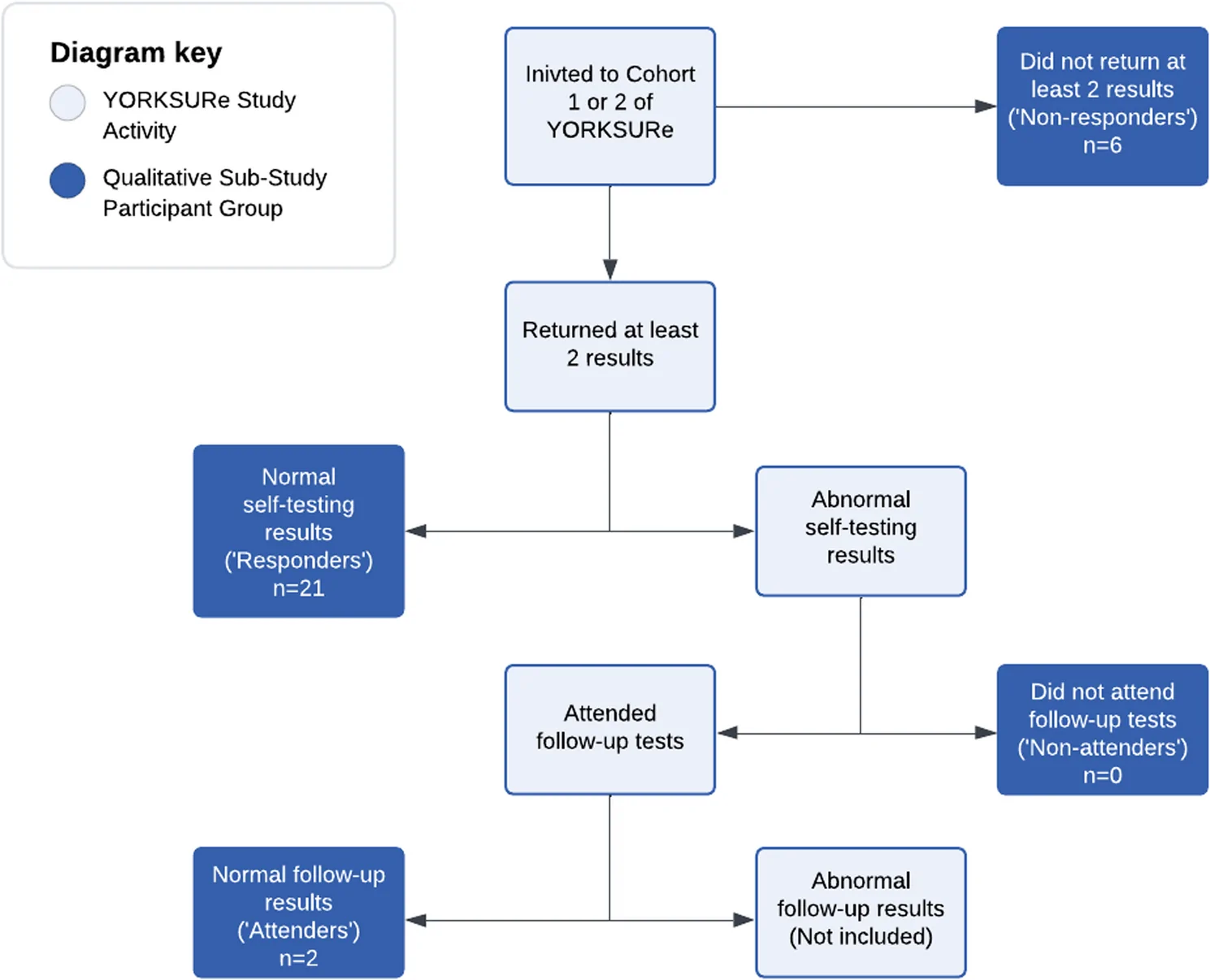
Participant groups for this qualitative sub-study of YORKSURe participants and non-participants

‘Responders’ were participants with normal self-test results (Fig. 1). ‘Non-responders’ were invitees who did not submit a self-test result. ‘Attenders’ were participants with abnormal self-test results who subsequently attended a follow-up appointment. ‘Non-attenders’ were participants with abnormal self-test results, who then declined or missed their follow-up appointment.

### Components evaluated

#### Urine self-testing kit

The kit included six test strips and an instruction booklet (Additional file 2, Image 1). The kit was described as a test to check for bladder health problems, including cancer. Participants were instructed to find a suitable container and conduct the test daily for six days. Participants could miss days but needed to complete at least two tests to receive a result. Urine could not be from the first morning urination, had to be ‘midstream’ and the test strip had to be dipped for 60 s.

#### Self-reporting results

Participants self-reported their results by phone or using a study-specific mobile app. To report by phone, participants called a freephone number with an automated system. Participants matched their test strip colours to those in a colour chart and entered the corresponding numbers (Additional file 2, Image 2). To use the app, participants started a 60-second timer, then the app automatically scanned the test strip. A nurse then reviewed the images to determine the presence of blood or glucose.

#### Reveal/conceal design

The test strips had five pads which tested erythrocytes (red blood cells), glucose, leucocytes, nitrites and protein. The result of only one parameter was reviewed by a nurse and revealed to the participant. In Cohort 1, this was haematuria. In Cohort 2, participants were randomised to either haematuria or glycosuria.

### Procedure

We invited potential participants to volunteer for an interview in reminder or result letters for the main study. Potential participants expressed interest by phone or email. Full information about the qualitative study was then provided to selected participants. Individuals agreeing to an interview provided consent prior to participation.

Interviews, expected to last about 30 min, were carried out by a female behavioural science post-doctoral researcher (EL) with training and experience in qualitative methods, following a semi-structured interview guide. There was no relationship between the interviewer and participants prior to the study. Only the name, role and organisation of the interviewer was provided to participants in advance. The interviewer’s position in a pro-screening research group may have shaped how some questions around screening intentions were asked and interpreted. Her personal background as a young female with a high level of formal education may have also influenced some participants’ willingness to speak candidly about their experiences collecting urine or their views on cancer screening more generally.

One-to-one interviews were conducted by phone and were audio recorded and transcribed verbatim. Field notes were kept during interviews to provide context to the data. Participants were provided £40 vouchers for their time. Transcripts were not returned to participants.

### Interview guide

The interview guide was informed by the Theoretical Framework of Acceptability (TFA) [20] and reviewed by two patient and public involvement (PPI) contributors who were men with lived BC experience. The TFA defines acceptability as ‘a multi-faceted construct that reflects the extent to which people consider an intervention to be appropriate, based on anticipated or experienced cognitive and emotional responses’. The framework includes seven constructs (Table 1). The interview guide addressed constructs relevant to each component under study, as well as barriers and facilitators to participation (Additional file 3). We conceptualised barriers and facilitators as issues inhibiting or encouraging participation related to the individual and their environment as opposed to the intervention alone.

**Table 1:** Definitions of the Theoretical Framework of Acceptability constructs

Construct	Definition
Affective attitude	How an individual feels about the intervention
Burden	The perceived amount of effort that is required to participate in the intervention
Ethicality	The extent to which the intervention has good fit with an individual’s value system
Intervention coherence	The extent to which the participant understands the intervention and how it works
Opportunity costs	The extent to which benefits, profits, or values must be given up to engage in the intervention
Perceived effectiveness	The extent to which the intervention is perceived to be likely to achieve its purpose
Self-efficacy	The participant’s confidence that they can perform the behaviour(s) required to participate in the intervention

### Analysis

Data was analysed using a combined inductive and deductive thematic approach to the Framework Method as described by Gale et al. [21] This includes seven stages:

1. Transcription.
2. Familiarisation with the transcripts.
3. Coding.
4. Developing a working analytical framework.
5. Applying the analytical framework.
6. Charting data into the framework matrix.
7. Interpreting the data.

Two researchers independently open-coded the first three transcripts to bring multiple perspectives into the analysis process, ensuring all important information was captured and examining how the information was interpreted. Researchers and the PPI contributors reviewed the codes and agreed a working analytical framework. Subsequent transcripts were coded by one researcher using the framework and then charted into a matrix. The research team members, including the PPI contributors, then interpreted the charted data and discussed the results. Participants did not provide feedback on the findings. All qualitative data was stored and managed in NVivo 14.23.1. The report was written following the consolidated criteria for reporting qualitative research (COREQ) guidance [22].

## Results

We interviewed 29 people in total, including 21 responders (72%), 6 non-responders (21%), 2 clinic attenders (7%). Roughly 2,081 people were invited to the YORKSURe Study during the qualitative sub-study recruitment period and 71 people who expressed interest. This included 45 people from Cohort 2 (63%) and 26 from Cohort 1 (37%), 51 responders (72%), 17 non-responders (24%) and 3 attenders (4%). The majority were male (55, 77%). No non-attenders were recruited as none volunteered. Interviews took place from January to July 2023 and lasted 12–55 min (average 22 min). As attenders were limited, we have not included results describing the follow-up test experience. Table 2 lists the participant characteristics.

**Table 2:** Participant characteristics

Characteristic	Number (%)
Participant status	
Responder (normal self-testing results)	21 (72%)
Non-responder (did not report results)	6 (21%)
Attender (abnormal self-testing results, underwent follow-up tests)	2 (7%)
Cohort	
1 (men & women)	10 (34%)
2 (men only)	19 (66%)
Random test group (only includes responders/attenders in cohort 2, *n* = 16)	
Haematuria	10 (63%)
Glycosuria	6 (38%)
Reporting method	
App	14 (48%)
Phone	8 (28%)
Neither	1 (3%)
Not applicable	6 (21%)
Age	
65–69	16 (55%)
70–74	6 (21%)
75–79	7 (24%)
Sex	
Male	23 (79%)
Female	6 (21%)
Ethnicity	
White background	29 (100%)
Education	
University	13 (45%)
Non-university	15 (52%)
Missing	1 (3%)
Sexual orientation	
Straight	28 (97%)
Gay	1 (3%)
Factory work	
Yes	17 (59%)
No	11 (38%)
Missing	1 (3%)
Cigarettes	
No	29 (100%)
Vapes / e-cigarettes	
No	26 (90%)
Yes	3 (10%)
Diabetes	
No	26 (90%)
Yes	2 (7%)
Missing	1 (3%)

When developing the analytical framework, the PPI contributors highlighted that views on result concealment had not been adequately explored, suggesting we lacked sufficient information power on this topic. The first 20 interviews had been conducted, so the interview guide was modified, and further interviews were undertaken for as long as resources allowed. We have separated the results by component of interest: Acceptability of self-testing, self-reporting and the trial design, understanding of the results, and barriers and facilitators to taking the test (Table 3). Direct quotes are included with their qualitative study ID, sex, participant group and age.

**Table 3:** Overview of thematic results

Trial component	Theme / sub-component	Sub-theme
1. Acceptability of self-testing	Attitudes toward urine self-testing	Confidence in the test The test kit Follow-up tests
	Experience of self-testing	Straightforward Burden / convenience Need for privacy
2. Acceptability of self-reporting	Self-reporting in general	Burden / convenience
	Self-reporting by app	No interpretation of results Accessibility and digital literacy Usability
	Self-reporting by phone	Capability Burden Usability
3. Acceptability of the study design	Randomisation	
	Concealment of results	
4. Experience of receiving the self-testing results	Inferring meaning from the test strip	
	Comprehension	
	Emotional impact	Waiting for results Receiving a negative (normal) result Receiving a positive (abnormal) result
5. Barriers and facilitators to the test	Timing Social influences Perceived need Attitude toward health Fear of cancer Free time Motivation	

### Acceptability of self-testing

#### Attitudes toward urine self-testing

Participants had generally positive views toward urine testing. They described being familiar with urine tests and liked that the test was ‘non-invasive’ (2-2345; male responder aged 69). No one expressed negative views toward urine testing.

##### Confidence in the test

Most people generally understood how the test might work (i.e. how colours change in the presence of certain molecules). However, participants seemed unconcerned about the specific mechanisms of the test.

‘How it works is difficult to understand… I thought I’ll leave it up to you experts.’ (2-2345; male responder aged 69).

Participants felt the sender gave them confidence in the test, highlighting either the NHS or the universities as trustworthy organisations. Some participants believed the organisations would only offer tests that work, whereas others were aware that the tests were part of research meaning they may not be effective. One person was reassured by the study’s ethical approval, while another felt reassured that clinicians were involved in interpreting the test strips. One person said the repeat tests increased their confidence, as collecting the samples over multiple days seemed more reliable than a single sample collected at a doctor’s office.

‘I trust you guys here, so I think if you obviously it’s a big research project … if it works, that’s brilliant. And you get all the results that you want. If it doesn’t work out, that’s how science is, and you learn from your mistakes and you move on.’ (2-3030; male responder aged 68).

‘I assume it could do, otherwise what’s the point of doing it? Why is the NHS spending all this money and giving me £40 worth of vouchers to do a test that’s irrelevant? I’m assuming that it has an efficacy, that it works.’ (2-2345; male responder aged 69).

##### The test kit

People described the kit as ‘well put together’ (2-3030; male responder aged 68), though a few expressed concern about the environmental impact of the plastic.

Several people felt the lack of container to collect the urine posed a challenge. Those concerned found it difficult to find a container deep and wide enough to collect the urine and match the test strip. A few people felt uneasy about urinating into a container that might be re-used. Others felt it was reasonable not to include a container as it would make the kit difficult to post.

‘I suppose finding a suitable receptacle for the urine, a container. I guess that was my only, I worried about what I should use, but anyway, I found a suitable glass, and then used that, and then threw it away at the end of the test.’ (2-3017; male responder aged 65).

Most people felt the instructions were clear and easy to follow. However, one non-responder reported he could not read the instructions, which was his main reason for not participating. He suggested the use of pictures like those in the bowel cancer screening kit would help people unable to read to take part.

##### Follow-up tests

Participants, including non-responders, were not surprised about the potential need for follow-up tests and were willing to attend further investigations for abnormal results. They saw follow-up tests as necessary to access treatment early for any medical issues. Some people felt the idea of further tests was ‘scary’ (1-1352; female responder aged 68) or concerning, but that ‘it’s great to have that security’ (1-1352; female responder aged 68) and reassurance from the tests.

Two people (one responder and one non-responder) were deterred by the potential need to travel for follow-up tests and another person said their caring responsibilities were a potential barrier to attending follow-up tests.

‘I could go to any test as long as I can get someone to be with my wife to make sure she’s okay.’ (2-3533; male responder aged 76).

#### Experience of self-testing

Most responders were able to complete the full set of 6 tests. Two people missed at least one.

##### Straightforward

Many participants were familiar with self-testing after COVID-19 and bowel cancer screening tests. Most participants, including non-responders, described the testing process as ‘straightforward’ and quick.

Some people thought the testing process seemed complicated on first impression, leading to delays in starting. People described the ‘inertia’ of doing the first test (2-2345; male responder aged 69) or a sense of hesitancy or ‘dithering’ (1-1204; female non-responder aged 73). They needed time to work out how to do the test and prepare but once people started, it became ‘easier and easier’ (2-2859; male responder aged 76).

‘After I did the first one I thought this is pretty straightforward. I don’t have to mess around too long, there is nothing taxing and I don’t have to do anything afterwards’ (2-2345; male responder aged 69).

The need to collect urine ‘midstream’ contributed to perceived complexity. While some people had no concern, many participants including responders and non-responders, felt unsure about what was considered ‘midstream’ or found it ‘messy’. One non-responder highlighted this as a key reason for not taking part.

‘Trying to work out midstream compared with the beginning and the end, was sort of, you know, sort of yes, with the best will in the world, it’s not confusing, I mean, yes, you’ve got urine, but at the same time, the midstream rather than the beginning and the end, didn’t help.’ (1-1536; female non-responder aged 77).

Most participants were indifferent about not collecting samples from the first urine of the day. Some responders saw this positively as they could complete the test flexibly at any other time. This included one responder who was initially apprehensive due to full-time caring responsibilities. For him, the ability to collect the sample at any other time ameliorated his concerns about being too busy in the morning. In contrast, some non-responders saw this as a constraint that compounded with the other requirements and added complexity. These non-responders who initially had negative reactions in the interview tended to change their minds after discussing the requirement in more detail, suggesting the instructions may not have been clear enough.

‘The thing about it as well, which makes it easier to do is that the urine sample that you were asked to test, it wasn’t to be the first urine sample of the morning, so because it was one that you could take any time during the day, that makes it easy, because you can do it at your own convenience. Whereas if you say, Oh, you’ve got to use your very first urine sample in the morning, that might, you know, cause people to run into issues.’ (2-2353; male attender aged 69).

##### Burden / convenience

People commonly reported that the test took ‘no time at all’ (2-3556; male responder aged 68) or less time than the instructions indicated. There were no negative comments about the time it took to complete the test. Many responders found completing the test at home convenient, avoiding doctors’ visits.

In contrast, some non-responders felt self-testing was more difficult than providing a sample in clinic. The need to repeat the test made it seem ‘involved’. They saw sampling over six days as challenging with other commitments like holidays or work trips. One non-responder felt the test seemed more burdensome than the bowel cancer screening test where you ‘do one swab, send it off, and that’s it, done’ (2-4326; male non-responder aged 66).

In contrast, responders highlighted that being able to miss days mitigated the burden of repeat tests. One responder who missed a day mentioned that if samples were required on consecutive days, they would have declined the test due to other caring responsibilities. However, responders did have trouble remembering to do the test or remembering if they had done the test already that day.

‘I have my grandchildren every week, so there was no way I could have a clear run at six days. The fact that within the packet stated it didn’t matter if you missed a day, was very reassuring. I felt well, it’s still, it’s worth doing then, you know? If it had said you have to do this six days on the trot, and you can’t miss a day. I wouldn’t have done it.’ (1-1352; female responder aged 68).

##### Need for privacy

This responder also highlighted that the ‘test was very simple’ but ‘you do need to allocate time, quiet time, to do it, and private time as well obviously.’ (1-1352; female responder aged 68) This person had caring responsibilities for children and worried about being interrupted.

### Acceptability of self-reporting

Fourteen participants reported their results by app, eight by phone and one person was unable to use either service. Two people who reported their results by phone initially failed to use the app and switched to the phone. The person who could not use either service first tried to report their results by phone.

#### Self-reporting in general

##### Burden / convenience

Some people viewed self-reporting as convenient. The sample did not need to be packed or delivered to a post office or clinic. Participants contrasted this to the bowel cancer screening test, where posting the sample was ‘a pain’ (2-3030; male responder aged 68).

‘I think [the bowel screening kit is] a lot more onerous than just doing it through an app where you just take a photograph and that’s it, it’s done.’ (2-3556; male responder aged 68).

One person found it is particularly useful given the test requires multiple samples, which would mean attending a clinic or posting samples repeatedly.

In contrast, some non-responders saw self-reporting, either by phone or app, as a challenge. These participants described self-reporting the results as an added burden on top of all the testing requirements.

‘The idea of doing this thing every day for six days. And the urine thing, and you had to do this, that, the other. Not the first one, second one, not the beginning, not the end, and then take a photograph of it and send it down the line. It was just too much for me to do, really. So that’s why I didn’t do it, you know? I mean, I would have liked to have done it. I believe in it. But I just felt I just couldn’t do it, you know? Well, I couldn’t do it easily, let’s put it that way.’ (1-1157; male non-responder aged 77).

‘To dip it in and then reading what it said on the dipstick, and then sort of I would have to then ring with the results and things because I don’t have the phone and computer facilities to do it otherwise. It was just everything that was involved in it that was sort of, well, you’re sort of thinking, blimey’ (1-1536; female non-responder aged 77).

Another participant saw self-reporting as a missed opportunity to see an expert. They preferred attending a clinic in person for the chance to see a clinician.

#### Self-reporting by app

Participants across the age-range described having mixed levels of digital literacy and access to mobile devices. Many participants expressed an initial preference toward reporting by app, perceiving the app as quicker, easier and ‘interesting’ (2-3028; male responder aged 69).

##### No interpretation of results

Many participants preferred reporting their results by app as this did not require interpreting the colours on the test strip. This included people who described themselves as having low to moderate digital literacy.

‘it’s easier for your phone to actually give the new people who are testing 100% correct evidence of what you’ve just, you know, the test you’ve just done. But you a person, an individual, might not give that correct information over the phone.’ (2-2859; male responder aged 76).

##### Accessibility and digital literacy

However, people also reported concerns about the accessibility of the app either for themselves or others. The spectrum of negative attitudes toward the app ranged from having concerns about *others’* ability to use it, having trouble using the app themselves, being unable to use the app due to digital literacy or device access (reporting by phone), to being deterred from taking part by mentioning an app (not participating).

Participants were concerned about others’ ability to use mobile apps due to age, cognitive ability (e.g. related to learning disabilities), eyesight and access to a device or the internet. Three non-responders cited concerns around the mobile app as a reason for not taking part.

‘it’s mostly the app thing and putting the information onto the app. That’s what puts me off.’ (1-1157; male non-responder aged 77).

‘Well, the main reason, I haven’t got an iPhone to, you know, when you’ve done what you’ve got to do with the strips. I haven’t got an iPhone. I’ve only got one of those cheap little things. I’m not on the Internet.’ (2-4308; male non-responder aged 78).

‘I mean it’s just the whole thing really, it’s just the multiple days, and having to report it online, which I wasn’t very good at, and I do recall now you’ve mentioned it about having to report it on the phone, I do remember that, but I’d already kind of made my mind up that I’ve got so much going on that enough was enough at the moment.’ (2-4326; male non-responder aged 66).

##### Usability

Most responders described the app as quick and easy to use. It had ‘no extras, no long explanations’ (2-2345; male responder aged 69). People expressed surprise at how easy they found it despite being ‘a little bit of a dinosaur with tech things’ (2-2715; male responder aged 69). People also described the additional features useful, like the video instructions and the timer. However, a few people mentioned the instructions for the app were unclear and a feature to keep track of the number of tests completed would be useful.

Despite the simplicity of the app, many people experienced technical issues. The most common issue concerned scanning the test strip and a few people had trouble finding and downloading the app. Participants reported trouble holding the phone steady enough to scan the strip or being unable to align it correctly p. This made one participant ‘very frustrated’ (1-1202; female responder aged 74), leading them to give up after attempting 4 tests, only 2 of which were successfully submitted.

‘So I put it to, you know, put it flat, I did what it said about, you know, not in bright light behind it, all that, I tried it on the floor, I tried it on the table, I tried it on the back, you know, and I could not get it. Couldn’t hold my phone steady enough to line it up.’ (1-1202; female responder aged 74).

#### Self-reporting by phone

##### Capability

Many people felt more comfortable reporting their results by phone, particularly those who described themselves as ‘not technology minded’ (1-0490; female responder aged 61). However, participants had mixed views on the average person’s ability to correctly report their results by phone.

Many participants felt confident they could match the test strip colours to the colour chart, including some non-responders. Others expressed concern. They felt people may interpret colours differently due to differences in perception, lighting, or eyesight. Some responders who reported their results by phone did experience difficulty. These participants felt the colours of their test strip did not match any of the squares on the chart, or that the colours on the chart were very similar, making it difficult to distinguish between the squares. The study initially had a technical problem where the booklet was printed incorrectly, however, the concerns persisted even after this was resolved. Both participants who were recalled for further testing had difficulty matching their test strip to the chart.

‘Well that’s open to ambiguity anyway I would imagine. Some people have different perception on colours.’ (2-4509; male responder aged 70).

Some participants were also concerned about correctly entering their answers into the phone system. They thought it would be easy to push the wrong button, making it vulnerable to mistakes. One participant did enter an incorrect answer and was unable to correct this using the phone system.

##### Burden

Participants who reported results by phone described the system as quick, but many who used the app perceived phone reporting systems as time-consuming. Some participants who preferred the app said they would have declined taking part if the results could only be reported by phone.

‘You know what it’s like these days making telephone calls. Quite often I’ve been doing this, you know, press keys for all these options and then hang on and listen to terrible music for 20 minutes. I would have been far more inclined to use the app.’ (1-1204; female non-responder aged 73).

##### Usability

Participants described the phone service as user-friendly. The feature saying the test number helped to keep participants on track.

‘I did it by phone because it was an easier option for me, but all the information, matching the colours and all that sort of thing, it was so simple to complete and very easy. And I was, you know, as I said, did it [inaudible] six times. So each time you did it, you got a little bit more confident and a little bit quicker.’ (2-3125; male responder aged 66).

### Acceptability of the study design

#### Randomisation

About half the participants from Cohort 2 remembered and reported understanding the randomisation. Participants generally accepted being randomly allocated to each group. Many participants preferred to receive both test results (haematuria and glucosuria) for personal benefit. However, most said they were familiar with this approach in research, making it seem more acceptable. Several people mentioned they were happy to receive at least one result compared to control groups where participants receive nothing. Some participants highlighted that the overall purpose of the trial was more important than choosing their group or having both tests.

‘I mean, if you turn around and said you get both, I thought that was great, but it wouldn’t put me off because something’s better than nothing.’ (2-4516; male responder aged 69).

#### Concealment of results

Concealment was mainly discussed in-depth with the last nine participants. When discussing concealment of the other four test parameters, many participants were surprised that more than one parameter was tested. Participants felt the information suggested only one parameter was tested.

Participants had wide-ranging views on concealing results. Some people felt strongly that all data should be collected and used to make the most efficient use of research resource regardless of whether participants could receive results.

‘if given that you, as I say, spent a lot of time and money and commitment doing all this stuff. I would have expected you to test for everything you possibly could, you know, in a way, it wouldn’t really matter if I got the results back or not. But it seems to me, if you’ve got all this data from us guys, it would make sense to use it all.’ (2-3030; male responder aged 68).

Participants who were happy only receiving one result mentioned either that one result was better than none or that the main purpose of the trial was more important.

In contrast, others felt that all results should always be shared. These participants expressed that more information was better for their own health. Despite this preference, some people said they would still take part in the trial despite not receiving all the results.

‘I would always want to know. If someone tests me for something, I would like to know the result. One way or the other.’ (2-4451; male responder aged 69).

In a few interviews, we further discussed how participants would have felt if the concealed results had been abnormal. These participants said they would expect to receive abnormal results. Some people accepted concealing these in a trial setting where the tests are not standard practice, while others expressed strong views that abnormal results should be shared regardless.

‘I think if anything is abnormal it should be reported back. It just gives the person the opportunity to take matters into their own hands and get it checked, and tested, and dealt with, you know? But if you’re just keeping it purely for research and not passing on any information, then that information is kind of lost until it develops into something better or worse.’ (2-4326; male non-responder aged 66).

### Experience of receiving the self-testing results

#### Inferring meaning from the test strip

A few people assumed they would receive multiple results based on the presence of five pads on the test strips or the fact that six test strips were needed. This meant some people were surprised to receive only one result. One person expressed uneasiness that the test strip might test for other conditions not mentioned.

‘I felt that maybe, I mean, I just have to keep my head on that it’s for cancer, not for anything else.’ (1-1352; female responder aged 68).

Several participants inferred their results based on the test strip colour changes and how this matched the colour chart.

#### Comprehension

Most people understood their result clearly. Participants appreciated the results being ‘very short and concise’ (2-2345; male responder aged 69). When probed on whether they would have liked further information, one person said they only wanted to know the results were normal.

#### Emotional impact

##### Waiting for results

Participants experienced a range of emotions while waiting for the test results, but none were described as severe. Some responders felt apprehensive, while others described being curious or interested. One person who reported having a ‘cancer phobia’ said they felt worried about the results. However, most participants said they had no concern. A few people attributed this to reassurance from other recent investigations or inferring normal results from the colours of their test strip.

‘I was interested to find out, but I wasn’t panicking or anything like that.’ (1-0500; male responder aged 70).

##### Receiving a negative (normal) result

Most participants who received negative results felt pleased. Participants who worried about the results reported feeling relieved. Others seemed more indifferent, framing the result as one of many tests being done ‘at that age’. Two people were surprised at receiving negative results given their history of haematuria.

‘The only thing I wanted to know, was everything was okay. So basically, I was happy with that. Yeah, I’m happy with that. There’s no problem. Carry on with your life basically.’ (1-1009; male responder aged 70).

##### Receiving a positive (abnormal) result

One person who tested positive reported feeling concerned as he had recently experienced bladder problems. He mentioned deciding not to disclose the result to his son and searching for potential causes online. However, he also described feeling pleased that something had been picked up that could be investigated and potentially resolved early.

In contrast, the other person interviewed who tested positive ‘half-expected’ (1-1383; male attender aged 72) the result as the colours of one strip had been different from the others. He did not describe any feelings of concern with the result.

### Barriers and facilitators to taking the test

#### Timing

The commonest barrier to uptake was inconvenient timing. Participants reported being away on holiday, experiencing a home crisis like a leaking pipe, or dealing with other health issues. All non-responders mentioned at least one timing issue. Some responders completed the test despite inconvenient timing, but non-responders felt the test was too much of an additional burden. Most non-responders said they would have tried the test if it had come at another time.

‘It was precisely the timing issue, because if you had sent me that kit a fortnight later or three weeks later, there would have been no problem. But I was obviously not going to do it while I had all this other stuff going on, which was actually very unpleasant. Horrible. So I didn’t actually want to think about, you know, collecting urine samples as well as everything.’ (1-1204; female non-responder aged 73).

#### Social influences

Social influences acted as both a barrier and facilitator. One non-responder explained that a friend had attempted the test but found it too messy and complicated. This deterred them from trying the test at all. In contrast, one responder described completing the test with their partner, helping each other make sense of the instructions and remember to do the test.

‘She actually started doing it and gave up because yeah, because she thought, yeah I mean, her reaction was, It’s just too messy and, you know, doing this and doing that, she said, I can’t be bothered.’ (1-1536; female non-responder aged 77).

#### Perceived need

Two non-responders reported having recent bladder scans with normal results. These tests provided reassurance so ‘there was less motivation’ (1-1204; female non-responder aged 73).

In contrast, a few people reported having recent or current urinary symptoms and saw this as an opportunity to check for related health problems. Two people who described having difficulty accessing health care saw the test as an avenue to check for issues while they waited for the standard tests.

I’ve had urinary problems, I’m seeing a urologist, supposedly seeing a urologist anyway… and then it all seemed to fall flat, even though I’ve been seeing the urologist. So I was very much looking forward to participating in this study.’ (2-2353; male attender aged 69).

#### Attitude toward health

The most common motivator was a general interest in checking for problems and maintaining health. Both responders and non-responders described being willing to take any health checks offered, particularly in older age. Most people understood the test as a general health check and seemed indifferent about the specific parameters tested for. Several people highlighted other areas of the body (e.g. the prostate or womb) the test might check. Some people suggested the purpose of the test ‘wouldn’t have made a difference’ (2-3030; male responder aged 68) as they were generally keen to screen for health problems. One person expanded on this saying they preferred getting some unnecessary tests and experiencing unnecessary anxiety than not knowing about a potentially serious health issue.

‘quite often I’ve heard people say the concern is that people become over worried and they might go and have further tests and in the end there’s nothing wrong. But I’d still prefer to be in that situation and something being wrong than not being aware of it’ (2-3030; male responder aged 68).

#### Fear of cancer

Some people reported being driven to take part by fear of cancer or cancer recurrence. One respondent initially felt apprehensive about participating after previously finding cancer through screening. The fear of the emotional impact of an abnormal finding initially deterred them from taking part.

#### Free time

Many people described being retired and having free time. Participants expressed an attitude of ‘why not’.

‘I’m retired, I’m footloose and fancy free.’ (2-3030; male responder aged 68).

#### Altruism

Most participants took part for altruistic reasons. People wanted to contribute to advancing science or improving the health of others. Many participants reported taking part in most studies when invited, even without personal benefit.

‘I think getting involved in studies is a good thing generally, to help science and to help medical science. And the future of your work and studies to make it better for people in the future. So I would try to get involved in most studies, if asked.’ (2-3628; male responder aged 70).

## Discussion

Overall, the idea of urine self-testing was generally acceptable. Many people felt capable of collecting and testing their urine samples and reporting the results. However, for some people, the stipulations required to collect the urine increased the perceived complexity and the need to self-report the results was an added burden. Further follow-up tests in the case of abnormal results were expected. The most common barrier to testing was timing, and the most common facilitators were general interest in health and past health experiences. There were mixed views on the acceptability of concealing results and concerns around concealing abnormal results.

Experience with COVID-19 tests and bowel cancer screening has increased comfort levels with self-testing. This post-pandemic window may be a prime opportunity to implement at-home tests as the public are receptive to this approach. Unfortunately, the test strips require several stipulations to ensure a reliable result (e.g. repeat tests over 6 days, midstream collection, not the first urine of the day). Modifying the test to reduce these stipulations would likely improve acceptability. Additionally, providing clearer instructions, such as describing ‘midstream’ and providing pictures to demonstrate the testing process, may improve uptake. Previous research has shown that providing illustrations can improve understanding of cancer screening [23].

While having two self-reporting options allowed more people to successfully submit results, challenges were encountered with both modes. Digital literacy and device accessibility deterred some people from reporting by app, while perceived capability to interpret the test strip colours discouraged people from reporting by phone. While national data suggests that mobile access is near ubiquitous, there is still a substantial proportion of people (14% age 55–64; 20% age 65+) without access to a smartphone [24. Even among those with smartphones, a proportion lack adequate digital skills or confidence to download and use the app. A recent report showed that 16% of UK adults lacked basic online skills, increasing to 45% among people over age 65 [25]. Even as the cohort with greater digital literacy ages, there will likely always be some groups excluded due to financial status, cognitive ability or mental health [26].

Providing an alternative reporting method by phone helps improve access. However, participants had concerns and difficulty interpreting the test strip colours, similar to a previous study among pregnant women [17] Other studies have acknowledged the potential limitation in reliability with urine dipstick self-testing [27]. Future work could investigate ways to improve the ability to distinguish between colours or improve the test strip to simplify interpretation. Non-responders in particular viewed self-reporting as an added burden. If urine testing is implemented in practice, it may be important to offer in-clinic testing in addition to at-home testing to ensure high and equitable uptake. Offering multiple methods of testing (e.g. self-sampling and clinician-sampling) has shown to effectively improve uptake in cervical cancer screening [28].

The need for privacy only appeared once in this work, but it may be more prevalent in practice. The participant reporting this barrier described being unable to complete the test while caring for grandchildren. Similarly, all non-responders reported that time and competing priorities contributed to them not taking part. Similar barriers were found among non-participants of the bowel cancer screening programme [29]. Trials have found that enhanced reminder letters lead to small increases in uptake of screening and slightly reduce differences in uptake by socioeconomic status [30]. Reminder interventions may also be effective in at-home urine testing by re-contacting individuals when competing priorities may have passed.

Participants had mixed views about concealing results. From an individual perspective, participants may benefit from investigating abnormal results. From a research design perspective, concealing the other parameters increases statistical power and reduces the necessary sample size. This reduces the time and resources needed to determine if this screening method is effective and, if effective, would enable earlier roll out to save more lives. From a clinical perspective, sharing results for non-standard tests could lead to unnecessary investigations or false reassurance. For example, a previous study found that at-home urine dipsticks failed to identify 85.9% of individuals with diabetes [27]. However, it also had very high specificity with 99.3% of participants with abnormal results diagnosed with diabetes.

Further consideration of revealing abnormal results for certain parameters may be warranted. Additionally, it should be reiterated at each relevant point that participants should contact their healthcare provider in the case of any symptoms. Explicit details about the parameters tested and whether abnormal results will be shared could be provided in future trials to ensure informed decision-making.

### Strengths and limitations

Although the sample was self-selected, the work was strengthened by including non-responders, as the views of participants likely differ from those who did not participate. Before starting recruitment, we expected very few non-responders to volunteer for an interview but recruitment greatly exceeded expectation.

We included a limited number of ‘attenders’ and no ‘non-attenders’ meaning we were unable to look at participant experiences of the follow-up tests. However, our results were reassuring as all participants expected and expressed an intention to attend follow-up tests in the case of abnormal results. Whether this intention translates to uptake of the follow up tests will be important to investigate in future.

The study sample lacked ethnic diversity limiting its generalisability, although this somewhat reflects the Yorkshire population where over 85% are from white ethnic backgrounds [31]. This is likely even higher among people over age 65. The sample was also skewed toward men, limiting generalisability further, although this was expected as Cohort 2 of the YORKSURe study included only men. The study did, however, include participants with a wide range of education levels and reading abilities which builds confidence that we included a range of perspectives. The £40 voucher may have helped reduce financial barriers and encouraged equitable participation.

Our investigation into attitudes toward the reveal / conceal design was unfortunately limited as this was only covered in-depth in the last nine interviews. Therefore, the conclusions on this topic should be treated as preliminary. However, this demonstrates the importance of PPI and strengthened the research in obtaining these important views to inform any future trial.

## Conclusions

At-home urine testing is a promising avenue for bladder health screening given its broad acceptability. The test evaluated in this sub-study was acceptable to many participants but could be improved by simplifying the urine collection stipulations and instructions. Self-reporting the results is convenient for some people but an added burden or challenge to others. Any future trial should consider providing clearer information about the parameters tested and the circumstances of any result concealment to ensure fully informed decision-making.

## Supplementary Information

Supplementary Material 1.

Supplementary Material 2.

Supplementary Material 3.

Supplementary Material 4.

## Data Availability

The coding framework is available upon reasonable request to the corresponding author.

## Abbreviations

BC: Bladder cancer
COREQ: Consolidated Criteria for Reporting Qualitative Research
COVID-19: Coronavirus disease 2019
ID: Identifier
ISRCTN: International Standard Randomised Controlled Trial Number
NHS: National Health Service
PPI: Patient and Public Involvement
TFA: Theoretical Framework of Acceptability
UK: United Kingdom
