# Supplementary material for: Acceptability of at-home urine self-testing for bladder health problems: a qualitative study
Source: BMC Public Health. 2026 Aug 12;26:3259. doi: 10.1186/s12889-026-28938-1 (PMC13746608; doi:10.1186/s12889-026-28938-1)
Supplement: Supplementary file 1 [file 12889_2026_28938_MOESM1_ESM.docx]

## Additional file 1. Sampling matrix.

### Sampling matrix for responders & attenders

Responders and attenders were be sampled consecutively until the quota for one of the cells in the sampling matrix was complete. After that point, only people who met criteria of the sampling matrix cells that are incomplete were be invited for an interview. People not invited for an interview were sent a holding letter which says they would be contacted if there are more opportunities to interview people before the end of the study.

|  | **Cohort 1**  **(Lung screening cohort)** | | **Cohort 2 – Haematuria**  **(GP invite cohort)** | | **Cohort 2 - Glycosuria**  **(GP invite cohort)** | |
| --- | --- | --- | --- | --- | --- | --- |
|  | **Responders** | **Attenders** | **Responders** | **Attenders** | **Responders** | **Attenders** |
| **IMD 1-5** | 1-2 | 1-2 | 1-2 | 1-2 | 1-2 | 1-2 |
| **IMD 6-10** | 2 | 2 | 2 | 2 | 2 | 2 |

### Sampling matrix for non-responders & non-attenders

Ideally, we wanted the mix of non-responders and non-attenders in the table below. However, we recognised that people who did not take part in the study were unlikely to be interested in an interview. We planned to include up to 6 people. If we had more interest than expected, we would attempt to balance following the matrix below. If there was limited interest, we would sample consecutively.

|  | **Cohort 1** | **Cohort 2** |
| --- | --- | --- |
| **Non-responders** | 1 | 1 |
| **Non-attenders** | 1 | 1 |
