# Supplementary material for: Acceptability of at-home urine self-testing for bladder health problems: a qualitative study
Source: BMC Public Health. 2026 Aug 12;26:3259. doi: 10.1186/s12889-026-28938-1 (PMC13746608; doi:10.1186/s12889-026-28938-1)
Supplement: Supplementary file 2 [file 12889_2026_28938_MOESM2_ESM.docx]

## Additional file 2. Self-testing kit.

**Image 1.** Self-testing kit.

**Image 2.** Colour chart.
