# Supplementary material for: Acceptability of at-home urine self-testing for bladder health problems: a qualitative study
Source: BMC Public Health. 2026 Aug 12;26:3259. doi: 10.1186/s12889-026-28938-1 (PMC13746608; doi:10.1186/s12889-026-28938-1)
Supplement: Supplementary file 3 [file 12889_2026_28938_MOESM3_ESM.docx]

**Additional file 3. Interview guide.**

**Introduction**

- Thanks for taking part, introduce yourself
- Double check consent

**Start recording**

- Explain the focus is on acceptability of the self-testing kit, potential follow-up tests and the study itself
- Reminder interview will last about an hour
- No right or wrong answers
- Reminder of confidentiality and ability to move on, stop or withdraw from the interview at any time.  Withdrawal can even be at the conclusion of the interview.
- Mention that we’ll end with a few questions about them
- First let’s talk about your thoughts on the self-testing kit, then we’ll take a step back and talk about your thoughts on the study itself. Then we’ll finish with a few brief questions on you and your health if that’s okay.

**1. Self-testing kit**

- *Open* can you tell me what you **remember about receiving the kit** in the post and your **thoughts about the kit**?
- *Affective attitude* **feel** when first saw the **kit**?
- *Self-efficacy* **understood** what to do to use the kit?
- *Self-efficacy* **confident** doing the test correctly?
- How many days did you complete the test?
- *Affective attitude* **feel about** doing the test?
- Anything that **put you off** using the kit?
- *Burden* did you feel the kit took considerable **effort**? Easy / difficult?
- *Opportunity costs* **miss something** to do the tests (i.e. because of the time it took)?
- Anything that made you **want to use** the kit?
- *Coherence* can you remember if you felt you **understood** the purpose of the kit?
- *Coherence* can you remember if you generally **understood how the test worked**?
- *Effectiveness* **confident** the kit could **find health problems**?

**2. Reporting results**

- *Open* **thoughts** about reporting in the app or by phone?
- Any **difficulties** **reporting results**?
- *Self-efficacy* **confident** using the **app or phone service**?
- *Self-efficacy* if reported by phone, **confident** correctly identifying the test strip **colours**?
- *Burden* how much **effort** to **report** each day? Easy / difficult?

**3. Receiving results**

- *Open* **thoughts** on receiving **results**?
- *Coherence* **understood the results** provided by the nurse?
- *Affective attitude* **feeling** receiving **results** of the test strips?
- *Ethicality* overall, do you see any issues with the kits and results in terms of your personal beliefs or preferences (i.e. political, social, environmental, religious, etc)?
- For example, Jehova’s Witnesses may reject blood transfusions for religious beliefs. Some people may reject certain cosmetic products that are tested on animals for animal rights beliefs. Is there anything about the self-testing kit that you think might be problematic?

**4. Further tests at your local clinic**

- Lots of information thrown at you, so it might have gotten lost, but do you remember mention of potential need for **follow-up tests** if positive? **If unaware skip to end of interview**
- *Open* **initial thoughts** on potential need for **additional tests**?
- *Coherence* did you understand the **purpose** of the potential additional tests?
- *Self-efficacy* would you have felt **confident attending** your local clinic for further tests?
- *Effectiveness* **confident** the further tests could **find potential health problems**?
- *Affective attitude* **feelings** about potential need for **additional tests**?

**5. Thinking back to your initial study invitation**

- *Open* Can you remember receiving the information in the post and your initial thoughts?
- *Open* Can you remember **why you decided to take part**?
- *Affective attitude* **feelings** about the **study**?
- Anything that put you off taking part in the study?
- Anything that encouraged you to take part?
- *Coherence* **understood** the aims of the **study**?
- *[Cohort 2] Coherence* **understood** the different groups you could be in and how you would be put into a group?
- *[Cohort 2] Affective attitude* **feelings** about the **randomised groups**?
- *Self-efficacy* **understood** what the **study** was asking you to do?
- *Effectiveness* **confident** in the study?
- Can you think of any ways this study could be improved?

**7. Closing**

- Few quick questions about you and your health behaviours – a bit of repetition, hope that’s okay. You can just tell me if you’d rather not answer any of them.
- Have you ever worked in a factory?
- Highest education completed?
- What is your ethnic group?
- Do you identify as LGBTQIA+
- Ever been diagnosed with diabetes?
- Do you smoke cigarettes?
- Vape / e-cigarettes?
- Thank you – any other comments or points you’d like to make?
- Reassure about confidentiality

**Stop recording**
