# Supplementary material for: Acceptability of at-home urine self-testing for bladder health problems: a qualitative study
Source: BMC Public Health. 2026 Aug 12;26:3259. doi: 10.1186/s12889-026-28938-1 (PMC13746608; doi:10.1186/s12889-026-28938-1)
Supplement: Supplementary file 4 [file 12889_2026_28938_MOESM4_ESM.docx]

**Additional file 4. COREQ Checklist**

| **No.** | **Item** | **Guide question / description** | **Reported** | **Where reported in the manuscript / notes** |
| --- | --- | --- | --- | --- |
| **Domain 1: Research team and reflexivity** | | | | |
| ***Personal characteristics*** | | | | |
| 1 | **Interviewer / facilitator** | Which author/s conducted the interview or focus group? | **Yes** | Methods – Procedure |
| 2 | **Credentials** | What were the researcher’s credentials? e.g. PhD, MD | **Yes** | Methods – Procedure |
| 3 | **Occupation** | What was their occupation at the time of the study? | **Yes** | Methods – Procedure |
| 4 | **Gender** | Was the researcher male or female? | **Yes** | Methods – Procedure |
| 5 | **Experience and training** | What experience or training did the researcher have? | **Yes** | Methods – Procedure |
| ***Relationship with participants*** | | | | |
| 6 | **Relationship established** | Was a relationship established prior to study commencement? | **Yes** | Methods – Procedure |
| 7 | **Participant knowledge of the interviewer** | What did the participants know about the researcher? e.g. personal goals, reasons for doing the research | **Yes** | Methods – Procedure |
| 8 | **Interviewer characteristics** | What characteristics were reported about the interviewer/facilitator? e.g. bias, assumptions, reasons and interests in the research topic | **Yes** | Results – Reflexivity |
| **Domain 2: Study design** | | | | |
| ***Theoretical framework*** | | | | |
| 9 | **Methodological orientation and Theory** | What methodological orientation was stated to underpin the study? e.g. grounded theory, discourse analysis, ethnography, phenomenology, content analysis | **Yes** | Methods – Analysis |
| ***Participant selection*** | | | | |
| 10 | **Sampling** | How were participants selected? e.g. purposive, convenience, consecutive, snowball | **Yes** | Methods – Participants |
| 11 | **Method of approach** | How were participants approached? e.g. face-to-face, telephone, mail, email | **Yes** | Methods – Procedure |
| 12 | **Sample size** | How many participants were in the study? | **Yes** | Results |
| 13 | **Non-participation** | How many people refused to participate or dropped out? Reasons? | **No** | Participants were not directly invited so this is unknown |
| ***Setting*** | | | | |
| 14 | **Setting of data collection** | Where was the data collected? e.g. home, clinic, workplace | **Yes** | Methods – Procedure |
| 15 | **Presence of non-participants** | Was anyone else present besides the participants and researchers? | **Yes** | Methods – Procedure |
| 16 | **Description of sample** | What are the important characteristics of the sample? e.g. demographic data, date | **Yes** | Results – Table 2 |
| ***Data collection*** | | | | |
| 17 | **Interview guide** | Were questions, prompts, guides provided by the authors? Was it pilot tested? | **Yes** | Methods – Interview guide and Additional file 3 |
| 18 | **Repeat interviews** | Were repeat interviews carried out? If yes, how many? | **Yes** | Methods – Procedure |
| 19 | **Audio/visual recording** | Did the research use audio or visual recording to collect the data? | **Yes** | Methods – Procedure |
| 20 | **Field notes** | Were field notes made during and/or after the interview or focus group? | **Yes** | Methods – Procedure |
| 21 | **Duration** | What was the duration of the interviews or focus group? | **Yes** | Methods – Procedure and Results |
| 22 | **Data saturation** | Was data saturation discussed? | **Yes** | Methods – Participants and Results |
| 23 | **Transcripts returned** | Were transcripts returned to participants for comment and/or correction? | **Yes** | Methods – Procedure |
| **Domain 3: Analysis and findings** | | | | |
| ***Data analysis*** | | | | |
| 24 | **Number of data coders** | How many data coders coded the data? | **Yes** | Methods – Analysis |
| 25 | **Description of the coding tree** | Did authors provide a description of the coding tree? | **Yes** | Results – Table 3 |
| 26 | **Derivation of themes** | Were themes identified in advance or derived from the data? | **Yes** | Methods – Analysis |
| 27 | **Software** | What software, if applicable, was used to manage the data? | **Yes** | Methods – Analysis |
| 28 | **Participant checking** | Did participants provide feedback on the findings? | **Yes** | Methods – Analysis |
| ***Reporting*** | | | | |
| 29 | **Quotations presented** | Were participant quotations presented to illustrate the themes/findings? Was each quotation identified? e.g. participant number | **Yes** | Results |
| 30 | **Data and findings consistent** | Was there consistency between the data presented and the findings? | **Yes** | Results and Conclusions |
| 31 | **Clarity of major themes** | Were major themes clearly presented in the findings? | **Yes** | Results |
| 32 | **Clarity of minor themes** | Is there a description of diverse cases or discussion of minor themes? | **Yes** | Results |
